# Professionals´ readiness for change to knowledge-based palliative care at nursing homes: a qualitative follow-up study after an educational intervention

**DOI:** 10.1186/s12904-022-01018-y

**Published:** 2022-07-20

**Authors:** Helene Åvik Persson, Gerd Ahlström, Anna Ekwall

**Affiliations:** Department of Health Sciences, Faculty of Medicine, Lund University, Box 157, 221 00 Lund, SE Sweden

**Keywords:** Educational intervention, Nursing home, Organizational readiness for change, Palliative care

## Abstract

**Background:**

There has been a global increase in the number of people who are dying of old age. This development implies a need for good palliative care among older persons at the end of life. Here nursing homes have an important role to play. However, the principles of palliative care have not been sufficiently applied in nursing homes, and there is a need to increase the implementation of palliative care in these settings. Therefore the project named Implementation of Knowledge-Based Palliative Care in Nursing Homes (the KUPA project, to use its Swedish acronym) was started as a contribution to filling this knowledge gap. The aim of the present study was to investigate the professionals’ experiences of readiness for change to knowledge-based palliative care at nursing homes after the educational intervention within the KUPA project.

**Methods:**

The focus group method was used to interview 39 health-care professionals with the aid of semistructured questions based on the Organizational Readiness for Change theoretical framework. Six focus groups were formed at six nursing homes in two counties in southern Sweden. The groups included different types of professionals: assistant nurses, nurses, occupational therapists, physiotherapists and social workers. The analysis was conducted with an abductive approach and included deductive and inductive content analysis.

**Results:**

The analysis revealed one overarching theme: hopeful readiness for change in palliative care despite remaining barriers. The main categories were increased knowledge facilitating development, enhanced team spirit, uncertainty about future plans connected with hopeful readiness and remaining organizational barriers.

**Conclusions:**

This study adds knowledge and understanding concerning professionals’ readiness for change palliative care in nursing homes and shows how ready nursing home settings undertake these changes in practice. The Organizational Readiness for Change theory proved suitable for application in nursing homes to assess the professionals’ experiences and to evaluate educational interventions regardless of the organization’s readiness for change.

**Trial registration:**

ClinicalTrials NCT02708498, first registration 15/03/2016.

## Background

The average age of the world’s population is increasing, and the human lifespan will continue to increase: the proportion of persons aged 65 or over is expected to increase from 9.3 per cent in 2020 to 16.0 per cent in 2050 [1–3]. Thirty-eight per cent of deaths in Sweden occur in a nursing home and 89% of all people who died in 2020 in Sweden were 65 or older [4–6]. A move to a nursing home or sheltered housing becomes necessary when an older person is too ill and frail to be cared for in their own home. Almost one-third of the older persons die within 6 weeks after moving into a nursing home [5]. A large proportion of frail older persons suffer in combination with ageing from various disabilities and diseases (multimorbidity) that imply a need for health care and social care. Disease trajectory in older persons is often prolonged and unpredictable, which can make it problematic to identify when the older person is dying [7]. Common causes of death in older persons are chronic diseases such as cancer, dementia and heart and circulatory diseases [5, 8]. The palliative care philosophy should enable access to palliative care as an integrated part of the care and should be present in every setting [9, 10]. Thus, long-term care facilities, such as nursing homes, play an important role in the palliative care of older people [11]. The principles of palliative care are applied in hospices and specialised care units but have not been sufficiently applied in nursing homes [12]. Therefore, there is a need to increase the implementation of palliative care in the transition from general care to a palliative care approach in nursing homes.

Successful implementation of evidence is influenced by the level of organizational readiness for change. The importance of establishing organizational readiness for change among professionals has been clarified in previous research [13–16], and one strategy for creating this readiness was explained in the Organizational Readiness for Change (ORC) framework developed by Weiner [17]. Organizational readiness for change is a relatively new concept in health care and nursing contexts. The term “readiness” can be defined as “a state of being both psychologically and behaviourally prepared to take action, i.e., willing and able” [17] p. 2. Organizational readiness for change is a multilevel construct and can be analysed at different levels, such as the individual, group or organizational level. Furthermore, the concept of organizational readiness for change contains three different core concepts/conditions: change commitment, change efficacy and contextual factors. Organizational members’ change commitment can be described as the members' joint decision to pursue the actions required for implementation. Change efficacy contains members' belief in the collective ability to organize and carry out the actions involved in the implementation of organizational change, and contextual factors affect organizational readiness for change. The organizational climate can be such a factor that can fosters organizational readiness [17, 18]. If the ORC is high, the members in the organization have a greater persistence to overcome barriers [19]. Health care organizations are undergoing massive transformations globally, which puts demands on members to be ready for, take action and adapt to successfully make sustainable changes [16, 18, 20, 21]. One reason that new programs and policies in organizations fail is that organizational readiness for change is not established by leaders [22].

Translating research evidence into practice is a challenge in health care. A systematic review [15] of ORC and its relationship with the process of change adoption showed that professionals’ readiness for change is crucial in the process of innovation adoption, where ORC can be an important determinant of success. Using evaluations with ORC can provide valuable information for planning and enable a possible revision in various educations [15]. A recently published focus group interview study [23] based on the ORC theory investigated the professionals´ expectations and preparedness to implement knowledge-based palliative care before the educational intervention showed that the professionals felt hopeful about what the education could achieve for them and the workplace. However, the professionals were also doubtful about the organization´s readiness to implement palliative care in everyday work [23].

Regarding long-term care facilities for older persons, a recent scoping review on implementation strategies of palliative care identified the need for further research to understand how the uptake of the implementation can be facilitated [24]. Increased attention needs to be paid to the evaluation of ORC among professionals after an educational intervention to revise and develop practical guidance for professionals in nursing homes and facilitate implementation in the workplace [23]. This study is an effort towards filling this knowledge gap, which is a part of the project named Implementation of Knowledge-Based Palliative Care in Nursing Homes (abbreviation in Swedish KUPA project) [25]. Therefore, the aim of this study was to investigate professionals’ experiences of readiness for change to knowledge-based palliative care at nursing homes after the educational intervention in KUPA project.

## Methods

### Design

A qualitative focus group design was applied in this study. Group interactions are used in focus groups as part of the method [26, 27]. The advantage of this method is that the participants’ knowledge and experiences can be explored through discussion and by asking questions that can reveal what, why and how they think in a specific way [28].

### Nursing homes in Sweden

In Sweden, older persons are supported by a policy [29] that provides care to enable these individuals to live in their own homes for as long as possible [30, 31]. The right to an apartment in a nursing home is based on an older person’s need for everyday care, as assessed by social workers in the municipality. The nursing home provides a homelike atmosphere and offers around-the-clock care [32]. All of the professionals working in nursing homes are employed by the municipality. The most common professionals are assistant nurses (ANs) and care assistants (CAs), which provide most of the care to older persons [8]. They can have one or two years of education in secondary school, including gerontology, geriatrics, and palliative care, while others have no training [33, 34]. Registered nurses (RNs) are also working in nursing homes and instructs and delegate nursing care and medical tasks to ANs, which can include administrating medication and redressing wounds. Other professions working with older persons are registered occupational therapists (ROTs), registered physiotherapists (RPTs) and social workers (SW). ROTs work with preventive and health-promoting initiatives such as activities of daily living (ADL) training, adaptation of housing and aid prescription. RPTs help people maintain or improve their mobility by training functions in daily life and prescribing certain mobility devices as crutches. Physicians work as consultants for nursing homes.

### The research setting

This study is a part of the evaluation of educational interventions in palliative care in the KUPA project, which is described in more detail elsewhere [25]. A total of 30 nursing homes from two counties in southern Sweden were included in the project. The educational intervention was workplace-based to enable dissemination to the entire work team. At each nursing home, a group of eight to ten professionals participated in the five educational seminars over the course of six months. Two experienced registered nurses led the seminars, and project meetings were held weekly to allow the seminar leaders to share questions among themselves and with the project leader. The educational seminars conveyed knowledge and skills that could be transferred into routine practice in the nursing homes across five themes: the palliative approach and dignified care; communication with next of kin; existence and dying; symptom relief; and collaborative care. The participating professionals received an educational booklet as study material before the intervention for use during and between the seminars [25, 35]. The booklet contained assignments to be completed between each seminar and were discussed in each subsequent seminar. The unit managers at the participating nursing homes had promised before the start of the KUPA project to nominate seminar leaders among their own professionals who participate in this education. After the project, the nominated professionals further spread the knowledge to their colleagues.

### Sampling and participants

Professionals were eligible to participate in this study if they worked in the nursing homes at the KUPA project and participated in the educational intervention [23]. Each nursing home had a delegated contact person who was informed about this focus group study and asked the professionals at their workplace about participation. To generate a wide range of opinions about professionals’ readiness for change to knowledge-based palliative care, representatives from all different professionals at the included nursing home were selected. The sample variation in terms of staff for care, social work and rehabilitation as well as for frontline management maximizes the exploration of different perspectives within the focus group setting.

The professionals in this study represented six nursing homes of different sizes located in different areas (rural/urban) and from both counties of the KUPA project [25]. A total of 39 professionals participated in the focus group interviews, of whom 37 were female and 2 were male. The participants included 28 AN, 6 RN, 3 OT, 1 PT, and 1 SW. One-third of the participants had a bachelor’s degree. Further background data were not collected.

### Data collection

Directly after the educational intervention was finished in the two counties, one focus group was performed in the participant workplace at each included nursing home. The six focus groups consisted of five to eight participants in each group. A moderator (M), a researcher with experience in leading focus groups, led the focus group interviews; an assistant moderator (AM), who was also researcher, was responsible for the digital equipment, observed the interviews and took notes. Before the interviews started, the purpose of the interview was again explained to the participants by the moderator. A semistructured interview guide based on ORC theory was used during the interview [17]. The following five broad questions were included in the semistructured interview guide:

- 1. What has the educational intervention in palliative care meant for the nursing home in which you work?
- 2. What are your thoughts on how palliative care education will be carried out after the KUPA project at your nursing home?
- 3. How do you currently experience the readiness for change within your organization?
- 4. How do you experience your managers´ readiness for change (i.e., to motivate and engage)?
- 5. In your opinion, what facilitates or complicates the implementation of palliative care?

The broad questions were followed with probing follow-up questions. The six focus group interviews had an average duration of 43 min (34–53). The digitally recorded interviews were transcribed verbatim.

### Data analysis

The data from the focus group interviews were analysed with an abductive approach, which included both deductive and inductive analyses. Abductive reasoning can be described as complementary and can be used to achieve a more complete understanding by moving between deduction and induction [36]. Abduction is a way to discover meaningful underlying patterns that makes it possible to integrate surface and deep structures. The analysis process can be described with an abductive approach because theory and empirics alternated [37].

### Deductive analysis

Deductive analysis is a concept-driven method where the implications of predefined concepts [23] were tested against the collected data and theory testing [38]. The analysis was initiated through transcribed interviews that were read several times to make sense of the data. Meaning units were identified in the interview text. Next, the deductive analysis was applied by the predefined concepts, which in this case are the findings from the focus group study before the educational intervention in the KUPA [23]. The predefined concepts from that study were a theme H*opeful but doubtful about the organisation’s readiness*, four main categories and ten sub-categories: Increased knowledge (Encountering next of kin, Safety in palliative care); Consensus in the team **(**Increased interprofessional communication, Space for reflection); Vision for the future **(**Opportunities over obstacles, Creating routines/guidelines, Trust in leadership); Insufficient resources and prioritisation **(**Financial resources, Parallel development work, Lack of time). This analysis revealed that all the concepts deriving from predefined concepts could be identified in the data, but the content of the meaning units was different from what it was in the previous study [23] except in the case of one concept, Parallel development work. Therefore, an inductive analysis took shape.

### Inductive analysis

A qualitative inductive content analysis was performed as defined by Hsieh and Shannon [38] (p. 1278) as “a research method for the subjective interpretation of the content of text data thorough the systematic classification process of coding and identifying themes or patterns”. The method can be described as a data-driven search for patterns to identify similarities and differences [38]. The first step in the inductive analysis encompassed the interview text from the deductive analysis not fitting the meaning of the predefined concepts. The meaning units within the particular main categories and sub-categories were read several times to obtain a comprehensive understanding of the content. The meaning units were coded and later merged with other codes that were similar and belonged to the same group. Second, the meaning units related to the predefined concepts used in the deductive phase that could explain the area of content were latently interpreted. The inductive analysis in two steps resulted in a nuancing of the predefined concepts.

### Analysis of underlying patterns

The abductive approach was finalized through an interpretation of the results from both the deductive and inductive analyses. The analysis process was continuously iterated between interpretation of the part and the whole to develop a deeper understanding of the result pattern in its context. This resulted in a verification of the nuances of existing theme, main categories and sub-categories.

The first author (H.Å.-P.) did the analysis supported by the 12^th^ version of NVIVO software [39] in the matter of grouping and sorting data into codes and categories under higher-order headings. NVIVO is used both in the deductive and inductive analyses. The coauthors (G.A., A.E.) took part in the analysis in such a way that they read all interviews and reviewed all categories during the analysis process. Discussion concerning the interpretation of the findings between the authors was ongoing until satisfactory consensus was reached. The similarities and differences in the results between the current and previous studies [23] will be the focus of the discussion section.

## Results

**Fig. 1:**
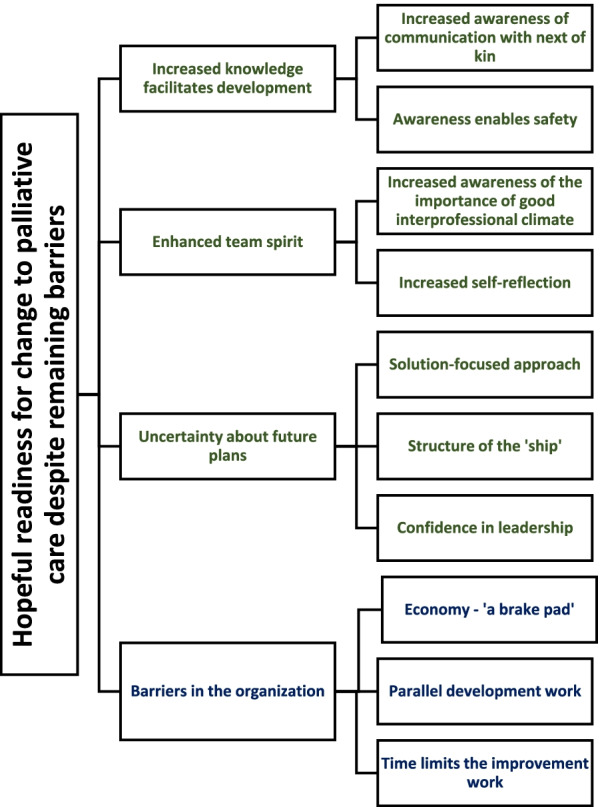
The results, as described in a theme, main categories, and subcategories. The main categories in green are connected with readiness and those in blue with the organisation´s barriers

The results consist of one theme, four main categories, and two or three subcategories in each main category (Fig. 1).

### Hopeful readiness for change to palliative care despite remaining barriers

The participants’ readiness for change in the organizations was expressed in their belief for future improvements to palliative care at the nursing homes. They looked forward to spreading further education to their colleagues, even though they considered it challenging to continue education and were hopeful about overcoming the organizational barriers. Another perception among the participants was that these improvements should be ongoing work because in a nursing home, palliative care is constantly present. However, there are organizational barriers to such improvements that experienced a challenge for the participants. Thus, participants’ positive attitudes towards overcoming barriers were widespread through the interviews and underpin the participants’ readiness to address palliative care in nursing homes.

### Increased knowledge facilitates development

Participants felt that they had obtained increased knowledge through the educational intervention, and thereby a readiness for change to knowledge-based palliative care. They described that they had a broader foundation to stand on after being educated, and increased attention to what they do in palliative care. ANs stated that they now had more knowledge of what signs they should look for before they contacted the RN. However, some participants, mainly RNs, reported that they felt that the intervention was too easy and that “hard facts” were missing. Other participants reported increased knowledge during and between the education sessions because there had been plenty of time for discussion and reflection. Participants also highlighted the creation of new structures and routines that were as a result from the education. According to the participants, it was valuable that new methods or tools became integrated in the daily work in palliative care, such as symptom assessment tools. The participants described that they valued not to becoming ʽ “jam-packed” with information during a short period as had happened in previous educational sessions. They appreciated the fact that this education had a different and improved pedagogical method, providing them with opportunities to reflect in groups over a longer period.

AN1: That is great because I mean it, then. Of course, I have known that you can use it [pain assessment], but I have never actually thought of using it myself or asking anyone about it.

AN2: I did not know that you (nurses) use the rating scale.

RN: No.

AN2: In that way, in addition, it is a great tool.

AN1: Yes indeed.

### Increased awareness of communication with next of kin

The participants expressed that they had become more aware of the importance of including next of kin. The importance of communication, such as informing next of kin of an older person’s deteriorating condition, is also something that was highlighted by participants of all professions. This awareness increased during the education; participants emphasized that exchanging experiences has given them insights into how to improve communication with next of kin. The participants emphasized the importance of “bringing next of kin with them during the palliative care journey”, as it considerably facilitates communication within the care process. However, the participants believed that it is important to obtain both positive and negative feedback from next of kin, as professionals can thus gain knowledge about what needs to be improved. Another aspect that was highlighted during the education is the discussion of bereavement support with next of kin, which takes place after the older persons death. ANs stated that they would like to be present during these conversations because it would be an opportunity to learn about the aspects that are highlighted during the dialogue between RNs and next of kin. They hoped that ANs would be more involved in these conversations and that this could be a routine at nursing homes. Difficult conversations were a subject that the participants considered would be part of the future education in the workplace. The participants considered difficult conversations as an important part of palliative care and they meant that they have received increased preparedness through the knowledge about this topic during the education. They hoped that they would have the courage to answer questions from both older persons and the next of kin.

### Awareness enables safety

The participants felt that they had received awareness and knowledge that could be helpful if difficult situations arose in palliative care. Increased awareness and knowledge about aspects that can happen near death in palliative care can lead to increased safety for the participants due to diminished fears in such situations. The participants saw that the consequences of having awareness in palliative care can increase the safety of meeting the older person in palliative care. Furthermore, feeling safe could enable a feeling of self-confidence and doing a good job among the participants. Another highlighted perception was that they now feel more prepared to answer questions from colleagues and the older persons that may arise in palliative care. The participants stated that they agreed to participate in this education due to fear of death; they hoped that the fears would be assuaged after gaining increased awareness, and they felt a higher level of safety after the education.

### Enhanced team spirit

The participants expressed that they had gained insight into the importance of cocreated care to improve palliative care, which promotes readiness for change within the organization. The team is important in palliative care, and the participants emphasized that trying to come up with solutions alone is not beneficial in this type of care. The participants stated that they had learned to focus more on the teamwork, which was something that needs to be constantly supported and under development. The discussions within the group during the education were helpful for the participants because they exchanged experiences and thoughts with each other. They also described an increased awareness of each other's professional roles, which the participants highlighted as a benefit of the education because everyone on the team had "building blocks" to add. The participants stated that everyone on the team complemented each other and that an increased understanding of each other's tasks had evoked the feeling that the team spirit would grow stronger during the education.

AN1: It is also an aspect that has been raised, this with our professional roles, that it is a closed circle that can be helped.

M: You can help each other?

AN: Yes exactly. That we have different building blocks to add.

RN: We complement each other.

M: Do you see your professional roles in a different way now?

AN: Yes, I actually think so and it can certainly increase it further and talk more about it.

### Increased awareness of the importance of a good interprofessional climate

The participants pointed out that to maintain the team spirit, it was necessary to lift each other up and praise each other whenever possible. Caring for each other on the team was an important part of good team results. Another aspect was that every member in the team should say what they think, and it should be a permissive work climate in the team. The participants believed that the education have make them more aware of what aspects of teamwork they were good at and what aspects needed improvement. Trying to erase the hierarchy within the team was also a reflection that had risen in the education. It was given that everyone should respect each profession, and someone's words should not be worth more than others' words. Another aspect highlighted by participants was listening to the colleagues and trying to be sensitive to everyone's needs, which were essential aspects of communication. OTs underlined that they experienced loneliness in their profession because they were only one OT per nursing home, but they also had an increased awareness about the importance of communication with the other members on the team. Additionally, the participants emphasized that communication between day staff and night staff needs to be improved. This was attributed to the isolation from joint activities with the day staff, such as workplace meetings, which resulted in a lack of knowledge among the night staff and a lack of consensus on the perspective of palliative care. The participants hoped to improve the interprofessional climate using continuing education.

### Increased self-reflection

The participants emphasized that the education had opened and raised thoughts in themselves that they might not have prioritized so highly otherwise. The participants meant that the seminar on existential issues aroused many emotions and made them develop their own thoughts about death and dying. They were given the opportunity to reflect on ​​existential thoughts about death that they felt they could carry with them in their professional role in palliative care. The participants thought that their self-reflection had increased in connection with the education and that it had been rewarding to hear about their colleagues' experiences. It stimulated their own reflection when they could exchange experiences and thoughts within the team. Achieved awareness about the significance of self-reflection has led to the possibility of continuing working with inadequacies in the workplace for the participants after completing education.

### Uncertainty about future plans

Uncertainty was expressed by the participants regarding whether there were any further plans from the management to continue improving palliative care. The immediate feeling was that there was a plan, but no concrete information regarding continued improvements had been presented to them by the organization. There was a will among the participants to continue their education in the form of study circles, making routines and developing structures in the workplace. Some participants had already begun formulating different thematic areas that they wished to include in future education. Other participants stated that they plan to use the material from this education and modify the content to adapt this to their workplace. The motivation to spread improvement work in palliative care through future education was high among the participants, which promoted readiness for change within the organization. The participants perceived that their managers were positive for continuing the education, but they were waiting for a clear message. Additionally, the participants had confidence in the leadership ability to develop a plan and believed that any barriers could be overcome.

M: Is there a plan for continued work with palliative care at your nursing home?

How do you and the manager think how you are going to get it together?

AN1: Yes, but I think she is interested in that.

AN2: Our manager seems motivated.

AN3: She has participated in the education herself.

PT: Must have clear goals and everyone must strives for the same goal.

RN: However, it appears to me that the manager has a plan.

AN1: Yes, it feels like that.

AN2: Yes.

RN: We do not know how she intends to involve all the professions.

AN3: No, we will see.

### Solution-focused approach

The participants expressed that it is usually possible to overcome the barriers that arise along the way, and it is important to change views and try to find solutions to the problems instead of giving up. The participants believed that their attitudes affect the ability to solve a problem. According to the participants' experiences, it is easier to solve problems if you have a positive attitude. An example was to create smaller groups during future education to decrease the need to bring in so many extra staff at each occasion. The participants believed that sometimes they needed to ‘think outside the box’ and see the solution in front of the problem. The participants highlighted the difficulties of getting all colleagues ‘on the train’ and experiences that patience and perseverance are required to carry out improvement work in the organization. The participants have experienced that changes can create resistance, and it is important to get everyone ‘on the same track’ and that everybody moves forwards together in the improvement work. The participants thought that it is important to market this education in a positive way to their colleagues when it will be disseminated further so that as many as possible are positive about participating. The demand for more education in palliative care had been great within the organizations, so the participants were hopeful that the dissemination would turn out well. They also stated that a large part of the improvement work in which they had been involved previously was based on shortcomings and not on what had been good in the organization. Other important aspects that were highlighted by the participants were that there must be time and patience for successful work with future education.

### Structure of the ʹshipʹ

Finding a structure in the team and developing routines was something that the participants planned to do now after the education was completed. They had a positive experience that different professions were represented during the education, as the participants’ main vision was to establish routines for the entire nursing home regarding palliative care that all professionals must adhere to. There was little time to raise these issues during working hours, and the participants thus considered this education valuable because the team had obtained the opportunity to discuss which routines were needed and how to design them. Some participants also believed that some existing routines need to be highlighted and discussed in the team, as these routines need to be updated or developed. One example was the inclusion of ANs in bereavement support, which was suggested during the education. Creating routines in palliative care was seen as something positive because everyone has a foundation to work from, which can also be applied when there are standbys. The fact that everyone works in the same way placed a great deal of emphasis on the participants.

### Confidence in leadership

There was a positive perception regarding leadership for the organization, and the participants had confidence in their frontline managers at the nursing homes regarding the initiation of future education and improvement work. The participants believed that the managers were good at boosting their creativity and were open to participation in the decisions when the opportunity arises. The participants were used to doing improvement work in the organization and pointed out that it is important that there is a functioning leadership and someone who controls the work. There is always someone who has negative attitudes towards improving work, and the participants believed that in these situations, the frontline manager has an essential role by influencing and encouraging everyone that is involved in the changes. The managers were open to ideas and suggestions, and the participants thought that the managers’ commitment to the educational intervention had been great. The participants thought that it was positive that the managers had participated in this education because they may prioritize future education if they are familiar with the content of the seminars. The participants were sometimes recognized for the work they did with rewards such as food or team activities. However, participants reported that the most important recognition was confirmation from either the older person, next of kin or colleagues that they were doing a good job. Many of the participants thought that their work was independent; they had the freedom to set up the work as they wished, and they felt that the manager trusted them. In addition, the managers were perceived as motivated and positive that improvement work is carried out in the workplaces. These qualities that the managers had shown made the participants confidence in the leadership.

### Barriers in the organization

According to the participants, the problem with the shortage of staff still remained as before the start of the education, which can negatively affect the organization’s readiness for change. It is difficult to obtain substitute and temporary staff, especially on weekends, which the participants believed hindered the organization from providing good palliative care due to the high workload during inconvenient working hours. The participants underlined that the work usually goes well from Monday to Friday, but there can be difficulties if a late palliative stage occurs on the weekend when an RN is needed, e.g., to give medicine for symptom alleviation. The participants considered that resources were an essential part of improvement work because it is impossible to carry out that if there is a lack of staff. Other problems that were raised in connection with the personnel resources that could affect the readiness for change within the organization were sick leave and staff turnover, mainly among the ANs and RNs.

M: Are there any barriers or problems that you can see that could arise when implementing a change to palliative care?

AN1: Yes, time and resources. However, everyone in the team is willing to work with this and has the will to change and would certainly work with it.

RN: Yes, I can imagine that. There must be time and resources to be able to sit down with the work as well. I think that is the biggest obstacle.

AN2: The greatest barrier I see is time.

### Economy – ‘a brake pad’

The participants were clear in emphasizing that the budget for their nursing homes were a barrier to continuing the improvement work in palliative care. They experience that everything that is done within the organization depends on the money. However, they believed that palliative care is such an important area that the budget should not be considered. The organizations in which the participants worked were constantly affected by cost-cutting measures, which created a feeling of inadequacy of never being able to have the opportunity to develop the work as they wanted. Another example was that the participants struggled to work with the existing budget, and then budgets were even further cut, thereby limiting their resources. The participants believed that these budget cuts had a negative effect on their commitment.

### Parallel development work

At the end of the education, there were still many ongoing parallel development works that were conducted and will be ongoing during the same time period as the continuing education in palliative care. However, all participants agreed that dissemination through future education should be a priority in the workplace. The ongoing projects in the workplace touched on areas such as dementia, documentation, mental illness in older persons and older persons´ health and quality of life. However, the participants believed that there was room for further development of the education in palliative care. Some participants did not truly understand how to get all the activities within the projects in their schedule because they felt overbooked.

### Time limits the improvement work

There was still a clear perception that time was one of the biggest barriers to continuing the improvement work in palliative care. The participants believed that this was a leadership issue, but at the same time, they were clear that enough time needs to be set aside to this work to obtain a long-term result. For some participants, it is about prioritizing what is the most important when there is much going on in the organization, and they also underlined the opportunity to prioritize. The participants were also aware that this work with palliative care would take time and something that they would have to work with for a long time. They hoped that the manager perceived palliative care as such a priority area and took the needed time into account in future plans for implementing improvement work.

## Discussion

This study reveals that the professionals had a positive attitude against the education and expressed a hopeful readiness for change in palliative care. Hopeful readiness involves knowledge and awareness, including benefits experienced with the implementation of palliative care, such as communication with next of kin, and professionals’ feelings of safety in care work with frail older persons. Another benefit that was experienced in connection with the education was that the team spirit was experienced as improved, which affected both the interprofessional climate and the professional’s self-reflection about palliative care. Furthermore, even if the professionals had an uncertain feeling about future plans, they were solution-focused, had confidence in the leadership and strived to maintain the structure of the ‘ship’. However, they experienced some barriers that remained in the organization at the end of the education, such as a lack of staff members, unsatisfied budget, competing parallel development work and not enough time.

### The findings before and after from the perspective of the ORC framework

We compared the current findings with predefined concepts from the focus group study before the educational intervention [23] and the ORC framework [17] (Table 1). In line with the concept of Change efficacy in the ORC theory, the professionals had expectations of increased knowledge about palliative care from the education, which they afterwards felt that they had indeed acquired. They had increased knowledge concerning different aspects of palliative care, such as communication with next of kin and safety, that did not exist before the educational intervention. To be aware of what is happening in palliative care contributes to safety and to understanding the importance of communication with next of kin is highly valued by professionals. In line with ORC theory [17], the professionals in this study have a belief in the collective ability to organize and carry out the planned actions to facilitate the implementation of education in organizations. Weiner [17] (p.4) stated three questions in connection with change efficacy: Do we know what it will take to implement this change effectively?; Do we have the resources to implement this change effectively?; and Can we implement this change effectively given the situation we currently face? If the members in the organization can respond positively to these questions, a sense of confidence that a change can be implemented is shared. These questions emphasize the importance of teamwork and connect to other findings in this study, which indicates that the participants perceived that they had learned to focus more on the collaboration and had increased awareness of each other's professional roles, which could strengthen the team spirit. The participants experienced that after completing their education, they had an increased awareness of the importance of a good interprofessional climate compared to before, where they called for better communication and a consensus on the team. Team climate has been described as the professionals’ shared perception of practices and procedures among the organization and describes the team members’ attitudes and behaviours [40–42]. Previous research [42] has highlighted the importance of a positive team climate to promote interprofessional collaboration. To endorse teambuilding, an understanding of the different roles among the professionals on the team was found to be valuable [43]. This is in line with the finding that the participants shared that taking care of each other within the team was an important part of achieving positive team results. Thus, it is important that time be given to support professionals in improving the team climate and collaboration over time [42]. The participants hoped to improve the interprofessional climate when the education should continue and be spread in the workplaces. This indicates that the participants had a change commitment and that readiness for change [17] was present among the participants and the organization.

**Table 1:** Overview of the three concepts of the Organizational Readiness for Change (ORC) theory and the main categories (in bold) and subcategories from study before implementation and follow-up study

**ORC **[17]	**Study before implementation **[23]	**Follow-up study**
*Change efficacy* Professionals´ belief in the collective ability to organize and carry out the planned actions involved in the implementation	**Increased knowledge** Encountering next of kin Safety in palliative care	**Increased knowledge facilitates development** Increased awareness of communication with next of kin Awareness enables safety
	**Consensus in the team** Increased interprofessional communication Space for reflection	**Enhanced team spirit** Increased awareness of the importance of good interprofessional climate Increased self-reflection
*Change commitment* Joint decision to pursue the actions required for the implementation	**Vision for the future** Opportunities over obstacles Creating routines/guidelines Trust in leadership	**Uncertainty about future plans** Solution-focused approach Structure of the ‘ship’ Confidence in leadership
*Contextual factors* Condition that affects organizational readiness for change such as resources, structure and culture	**Insufficient resources and prioritisation** Financial resources Parallel development work Lack of time	**Barriers in the organization** Economy – a brake pad Parallel development work Time limits the improvement work

The participants felt uncertainty in future plans with the improvement work (Table 1). Already before the education began, the participants had visions for the future to disseminate and spread the education to their colleagues. They had expectations before the education that this should be a living project. To succeed with the spread of the innovation, Berwick [44] suggests that simplifying the change makes it as easy as possible to disseminate. This was illustrated by the participants when they spoke about subjects they wanted to focus more on in the future education and spread to their colleagues. This finding indicates the professional´s change commitment in ORC theory [17], which includes the collective decision to pursue the action involved in change implementation. Herscovitch and Meyer [45] talk about three different types of commitment in regard to organizational change: want to, have to or ought to. The participants in this study “want to” make a change, which reflects the highest level of commitment regarding implementing a change [45]. In recent research [13], ORC indicated successful dissemination of evidence-based guidelines into health care, which can strengthen this study’s findings that the theory can be used to tailor and improve the dissemination of interventions. However, the feeling was that the managers had a plan for this further improvement work, but nothing concrete had been presented to the professionals. It is not uncommon that professionals can feel uncertainty about the change in an organization during different stages in an implementation, including a variation in the attitudes towards change [46]. Thus, they saw the solution in front of the problem and were open to continued improvements partly due to confidence in the leadership. In this study, leadership was not seen as a barrier either before or after the intervention. Similar findings were identified in the Nilsens study [18], which explored readiness to implement evidence-based palliative care in nursing homes from the managers’ perspective. Hence, leadership plays an essential role in change readiness [47], and from the ORC perspective [17], this positive attitude from professionals about solution-focused approaches and confidence to leadership can be concluded to be high commitment.

The barriers identified before the educational intervention [23] remained after the intervention (Table 1). The participants still thought that there was a problem with finding personnel resources in the nursing homes, which limited the possibility of implementing palliative care. A lack of staff demands a higher workload for professionals, which can affect professionals’ readiness for change. This is in line with the Nilsens study [18], who stated that resources and time were barriers to implementing evidence-based palliative care. Similar findings were identified in Millis’s [48] study, where a barrier in the implementation process was finding time. It was underlined that it was difficult to find time to complete activities and to complete ongoing training after the intervention. Another study by Zhao [49] emphasized that lack of resources and time were main barriers and made it hard for professionals to further develop their work. The findings in this study indicate that the participants have hopeful readiness for change palliative care, but there are still some barriers that can be seen as contextual factors in ORC theory [17]. Contextual factors are an important part of the organizational readiness for change, but there is a lack of research that identifies these factors, which in turn can affect the success of a change [50, 51]. The fact that the barriers remain after the intervention can be a sign that deeper change in the nursing home culture is needed. These barriers may be a part of the culture in the nursing home, which can be a challenge to change. A literature review [52] concerning cultural changes in nursing homes stresses that it is an evolving field, and there is a lack of evidence that provides guidance to implement changes in this field. To achieve a cultural change, policy-makers and providers must develop strategies to enable adaptation to practices. Resources within a nursing home are a factor when it comes to the facilitation of cultural changes [53]. The findings connected with the barriers in this study contain difficulties with available resources; however, the participants are still positive and believe that it is still possible to implement knowledge-based palliative care with current assets. This gives hope for a readiness for change among the participants in the organization.

### Clinical implications and future education

The findings highlight the importance of participants having readiness for change when a change is going to be implemented in the organization. Knowledge about these components that contribute to readiness for change is valuable both in the design phase and during and at follow-up to address the barriers and facilitators in the implementation process. Both geriatric and palliative care expertise is needed within the nursing home context, and this educational intervention is an attempt to enrich competence within professionals. However, to draw conclusions about long-term effects, it would be beneficial to make several evaluations after the educational intervention, preferably after six months or one year. These evaluations should also include experiences and perceptions of older persons and next of kin. Another factor that needs to be studied is the leaders´ perceptions of the ORC at follow-up.

The greatest increase in palliative care needs will be among older persons [54], who suffer in connection with ageing, from various disabilities and illnesses, which can lead to difficulties in identifying when ageing persons’ general care needs require transition to a palliative care approach [55, 56]. This gives rise to a need for increased education among professionals at nursing homes, which this education intervention provides the opportunity for and which, according to this study’s findings, provides increased knowledge about palliative care. The participants had a positive attitude both before and after the educational intervention, which expressed that they saw opportunities before obstacles and had a solution-focused approach. Dissemination of the education intervention can be a challenge, and it is important that the participants think that this can help them. Berwick [44] highlights several possible adopters regarding dissemination, and one of them is the perceived benefit of the change that is going on in the organization. The participants clearly exposed that they understood the benefits of this education and were uncertain but positive for the future.

### Methodological considerations

There are both limitations and strengths that need to be acknowledged in this study. The dropout rate compared with the focus group study before the intervention was 9 of 48 participants [23]. There were 6 dropouts in one county compared to 3 dropouts in the other participating county. The participants who dropped out held the following professions: four assistant nurses, one registered nurse, one occupational therapist, one physiotherapist and two social workers. There was a maximum number of two dropouts per focus group, which was assessed to have only a small influence on the results, and the data generated from the interviews consisted of 5–8 participants after the dropouts. According to Kreuger and Casey [26], a focus group should include 3–12 participants.

The majority of the professionals in elderly care in Sweden are women, which is in line with the gender distribution in the focus groups in this study. This distribution contributes to the fact that the result cannot be generalized to men but is limited to women. There were few participants with academic bachelor’s degree, which indicated a certain caution when transferring the results to other contexts, such as specialist units in palliative care, where more professionals have higher level of academic training. Although there is only information about gender and profession, this background information is perceived to be sufficient.

One limitation in the study is the length of the interviews, as the average length was 43 min. A few interviews did not take long, as some participants did not expand upon their answers, despite attempts by the moderator with follow-up questions. There was wide variation in how in-depth answers were, which affects the length of the interviews. This can affect the credibility because some participants did not detailed answers, and there were two interviews that were slightly shorter. However, there were participants in these shorter interviews who gave exhaustive answers that made the data feel saturated when the interview ended. Credibility was upheld through investigator triangulation [57].

Another limitation is that the follow-up focus groups were performed directly in connection to the end of the education. The purpose of this was to avoid requiring the organization to set aside additional work hours to complete the focus groups. This was intended to respect the lack of resources in the nursing homes we studied [23]. Although the follow-up interview took place close after the last education session, the focus group was an opportunity to reflect with work peers on the education as they had the content fresh in their minds. It can also be considered a limitation that the result in this study is based on only one follow-up that was made immediately after the intervention.

A strength of this study was the abductive approach because it enabled us to discover meaningful underlying patterns and to integrate surface and deep structures by changing between deductive and inductive approaches [37]. With the abductive approach, it was possible to apply the predefined concepts that emerged in the previous focus group study [23]. The existing knowledge is used to find theoretical patterns, which provides relief from being neutral from preconceptions. The approach also provides an opportunity to stop at the unexpected, as the knowledge building of everyday life takes place, which also gives room for spontaneity and intuition. Karlsen et al. [36] believes that with the help of the stepwise process in the abductive approach, it can inform nursing researchers and help to create evidence-based knowledge in nursing practice. Abduction is a research method that is based on discovering the unexpected, reconciling it with existing knowledge, and creating a new understanding that is then tested through new observations in a living and constantly ongoing process. However, a few articles using qualitative content analysis demonstrate the abductive approach, although it can be beneficial for the development and translation of evidence-based knowledge. Therefore, the method is recommended for nursing research in the future [58].

## Conclusions

This study has increased the understanding of professionals’ experiences regarding readiness for change in palliative care in nursing homes and showed how prepared nursing home settings are to undertake changes to achieve desired practice change. The professionals’ readiness for organizational change in this study can support implementing the educational intervention and enabling a sustainable change in palliative care. Based on the results of the study, ORC is a theory with three essential components that are suitable to apply in the nursing home context to capture professionals' experiences of readiness for change. According to the findings in this study, professionals have beliefs in the collective ability to organize and carry out the actions and have made the decision to pursue the actions involved in the implementation. Despite the organizational barriers, the professionals were solution-focused and positively focused on the future. Thus, there is a need for further evaluations after educational intervention at several intervals to follow the long-term significance for professionals, leaders, older persons and the next of kin.

## Data Availability

The data in the focus groups has a comprehensive content and the participants was not asked to permit public publishing. The datasets used during the study are available from the project leader, co-author (G.A.) upon written request and in accordance with ethical approval.

## Abbreviations

AM: Assistant moderator
AN: Assistant nurse
KUPA: Implementation of Knowledge-Based Palliative Care in Nursing Homes
M: Moderator
ORC: Organizational Readiness for Change
OT: Occupational therapist
PT: Physiotherapist
RN: Registered nurse
SW: Social worker
UM: Unit manager
